# Integrity of a HPV11 infection cell model and identification of (-)-Epigallocatechin-3-gallate as a potential HPV11 inhibitor

**DOI:** 10.18632/oncotarget.9249

**Published:** 2016-05-09

**Authors:** Yang Sun, Xinyu Li, Shasha Song, Yongfang Wang, Heng Gu

**Affiliations:** ^1^ Institute of Dermatology, Chinese Academy of Medical Science and Peking Union Medical College, Nanjing, China

**Keywords:** condyloma acuminatum, HPV11, early genes, IFN pathway, model

## Abstract

**Background:**

Condyloma acuminatum (CA) is one of the most common sexually transmitted diseases and induced by low-risk human papillomaviruses (HPVs), mainly HPV type 6 and 11. Here, we report the identification of (-)-Epigallocatechin-3-gallate (EGCG) by an HPV11 infection cell model.

**Results:**

The recombined HPV11.HaCaT cells had stable HPV 11 early genes expression. The introducing of HPV11 genome significantly increased the proliferation of HPV11.HaCaT cells, as well as the proportion of cells in S and G2/M phases. After treated with rhIFN-α 2a, IFN signaling pathway was activated in both HaCaT and HPV11.HaCaT cells, while HPV11 decreased the activation level. In addition, rhIFN-α 2a, could inhibit expression of HPV 11 E6 and E7 mRNA significantly (P<0.05). However, cell growth and cell cycle did not show statistical difference (P>0.05). Nevertheless, EGCG, a major active constituent in tea polyphenol, showed strong anti-HPV11 effect, which inhibited HPV11 E6 and E7 mRNA.

**Methods:**

Gene transfection technique was used to introduce HPV11 genome into HaCaT cells, named HPV11.HaCaT cells. With the established cell model, we explore the anti-HPV11 effect of (-)-Epigallocatechin-3-gallate (EGCG) on cell growth, viability and affection on expression HPV11 E6 and E7 mRNA.

**Conclusion:**

Our data collectively demonstrated that the recombinant HPV11.HaCaT cells were integral and practical to be a cell model to test anti-HPV11 agents and explore the interaction between HPV11 genes and host cells. And EGCG inhibits expression of HPV11 E6 and E7 mRNA in the recombinant HPV11.HaCaT cells.

## INTRODUCTION

HPVs cause a number of diseases ranging from benign lesions to malignant tumors. According to different pathogenicity, HPVs can be simply divided into “high-risk” groups (HR HPVs) and “low-risk” groups (LR HPVs). HR HPVs, mostly HPV 16 and 18, have been extensively investigated which are correlated with cervical cancer. HR HPVs can also lead to squamous carcinoma of vulva, penis, anal and oropharynx. Moreover, HPV16 infection might be a risk factor for prostate cancer [1]. Comparing to HR HPVs, LR HPVs mainly induce benign lesions, such as verruca vulgaris, flat warts and condyloma acuminatum (CA). Nevertheless, LR HPVs are correlated with clinical outcome of oral cavity squamous cell carcinoma [2]. CA, which mainly caused by HPV type 6 and 11, is a common sexually transmitted disease and the incidence is rising year by year all over the world. Drugs used locally for CA, including imiquimod, interferon, podophyllotoxin, etc., are focused on immunomodulating or cytotoxicity, not against HPVs. In addition to increasing incidence, the high recurrence rate of CA has also makes the disease become one of the common sexual transmitted disease affecting the public health problems. Application of medicines which directly target pathogens, including reducing the activity of HPVs or killing the virus, and affecting the virus replication or expression of a functional gene, will be one of the ideal therapeutic strategies of CA.

Human papillomaviruses (HPVs) are small double-stranded DNA viruses. There are more than 180 characterized HPV gene types, and new virus types are being continuously identified [3]. The host of HPVs is human mucosal and skin epidermal cells. HPVs encode eight open reading frames (ORFs) including early genes (E1, E2, E4, E5, E6 and E7) and late genes (L1 and L2), along with one non-coding long control region (LCR) or upstream regulatory region (URR). E5, E6 and E7 are oncogenes [4, 5]. Early genes are important in HPVs replication and oncogenes are responsible for the pathogenicity. HPVs genes, especially early genes, interact with host cells and result in a series changes in hosts. That induces the continuous infection status of HPVs and occurrence of diseases.

The challenge of developing specific anti-HPV agents is to build effective screening system to test specific anti-HPVs activities vitro and vivo. HPVs only replicate and complete their life cycle in human, and it's difficult to induce specific lesion at epithelial sites in vivo. Although the human epithelium xenograft immunocompromised mouse system has been reported, applying this mouse model as a screening and evaluating tool of anti-HPV agents is inconvenient because of its technically complexity and time-consuming process [6, 7].

Cell culture systems in which infection was mimicked by harbouring HPVs’ gene segments or whole genome are expected to be as a convenient screening model of anti-HPVs agents in vitro. Previously, we have developed a basal-like HaCaT keratinocytes containing genome of HPV 11 for screening of anti-HPV 11 effect, and proved stable replication of HPV11 DNA in definite passages of cells by detecting HPV11 DNA copy number and E1^E4 transcripts [8]. Because the complete life cycles of HPVs depends on the differentiation program of infected epithelial cells, we also observed that L1 capsid protein expression of HPV11 in the raft cultures of the recombinant cells [9]. As a mimetic model system for using to test anti-HPV effects, we have to bear in mind whether there was fully expression of functional early genes of HPV11 in recombinant cells harbouring HPV11 episomes and the impact on cellular microenviroment by virus genes stimulation.

Here, we further confirmed several early genes expression of HPV11 and observed the growth characteristics of the recombinant cells (HPV11.HaCaT). Meanwhile, using the recombinant cells system, we also tested the anti-HPV11 activities of (-)-epigallocatechin-3-gallate (EGCG), which is one of the important active ingredients of tea catechins. Sinecatechins (in a 15% ointment) extracted from green tea has been approved by FDA for the topical treatment external genital and perianal warts (condylomata acuminata). Also, we detected anti-HPV11 activities of recombinant human interferon-α 2a (rhIFN-α 2a) which has been used in the treatment of CA in clinic.

And then, we detected the IFN signaling pathway of cells after harbouring HPV11 episomes. Because keratinocytes are the natural target cells of HPVs and have immune activity. When HPVs infecting keratinocytes, cells can sense the pathogens and mediate immune responses. Acting as a bridge between innate and adaptive immunity, IFN response can be activated after HPVs infection [10]. Most DNA viruses, including HPVs, are capable of inhibiting IFN synthesis and IFN signaling pathway during maintaining infection [11]. It has been reported that HPV 18 E6 and E7 can inhibit the interferon response through JAK-STAT pathway [12, 13].

## RESULTS

### Expression of HPV11 early genes in the recombinant cells

**Figure 1:**
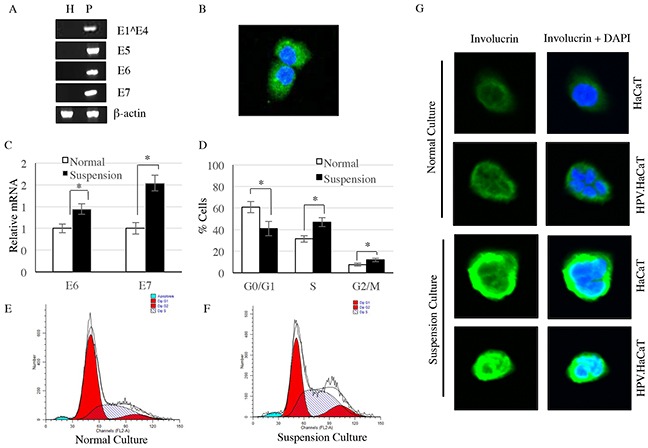
HPV11 early genes were identified in HPV11.HaCaT cells, but not HaCaT cells **A.** HPV11 early genes were only expressed in HPV11.HaCaT cells, while β-actin were expressed in both cells. **B.** Immunofluorescence showed E7 protein was expressed in HPV11.HaCaT cells. Green fluorescence indicated E7 protein and blue fluorescence indicated cell nucleus. **C.** The expression of E6 and E7 genes were increased significantly in suspension culture HPV11.HaCaT cells comparing to normal culture. **D–F.** In suspension culture HPV11.HaCaT cells, the proportion of cells in G2/M and S phases is significantly increased. **G.** The expression of involucrin is significantly increased in suspesion cultured of both HPV11.HaCaT and HaCaT cells, comparing with normal culture. Data are means ± SD from three independent experiments. H refers to HaCaT cells and P refers to HPV11.HaCaT cells.

Early genes were the main functional genes controlling replication and pathogenicity of HPVs. We firstly tested the early genes expression of HPV 11 in the recombinant cells which we called HPV11.HaCaT cells (Figure 1A). Since E1^E4 are sequential in structural, and have overlapping regions, nested PCR has been used to identify E1^E4, while E5, E6 and E7 were detected using reverse transcript PCR. In the recombinant cells, HPV11 early genes (E1^E4, E5, E6 and E7) were clearly expressed, but not in HaCaT cell line (Figure 1A). To explore the translation of HPV early genes into proteins, related proteins were detected by commercialized antibodies against HPV11early genes, i.e. anti-HPV11 E7 antibody. Immunofluroscence showed that HPV11 E7 protein could successfully express in the recombinant cells (Figure 1B).

### Expression of HPV11 E6 and E7 mRNA in differentiated recombinant cells

Growing suspended in 1.6% methylcellulose, HPV11.HaCaT cells tended to differentiate. We observed that there was a significant increase in the proportion of cells in G2/M and S phases when recombinant cells were cultured inmethylcellulose semisolid Media by a suspension manner (Figure 1D–1F). In the differentiated HPV11.HaCaT cells, HPV11 genes replicate rapidly. The differentiate marker involucrin was detected by immunocytochemistry, and the expression of involucrin was significantly increased in suspension cultured of both HPV11.HaCaT and HaCaT cells, comparing with normal culture (Figure 1G). Meanwhile, the expression of HPV11 E6 and E7 mRNA were increased significantly in differentiated HPV11.HaCaT cells compared with undifferentiated cells growing in liquid media (conventional culture manner) (P<0.01) (Figure 1C).

### Growth characteristics of the recombinant cells

**Figure 2:**
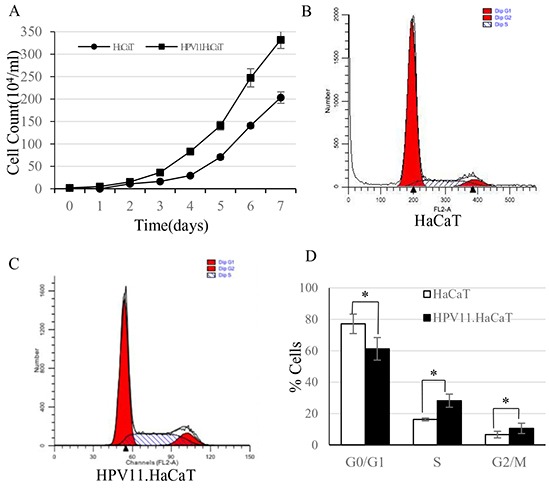
The cell growth curves and FACS analysis of HaCaT and HPV11.HaCaT cells **A.** Cell growth curve showed that HPV gene could promote the host cells’ proliferation. From the 3rd day, HPV11. HaCaT proliferated more rapidly than HaCaT (*P*<0.05). **B–D.** Cell cycle analysis in HaCaT and HPV11.HaCaT cells. In HPV11.HaCaT cells, there is a significant increase in the proportion of cells in G2/M and S phases (G1: HPV11.HaCaT<HaCaT, *P*<0.01; G2: HPV>HaCaT, *P*<0.05; S: HPV>HaCaT, *P*<0.01).

The growth curves showed that the HPV11.HaCaT cells could proliferate more rapidly than HaCaT cells (Figure 2A). Flow cytometry showed the proportion of cells in G1 phase was decreased in HPV11.HaCaT cell line, with an increase in S and G2/M phase compared with HaCaT cells (Figure 2B–2D; *P*<0.01).

### The effect of rhIFN-α 2a on the infection cell model

**Figure 3:**
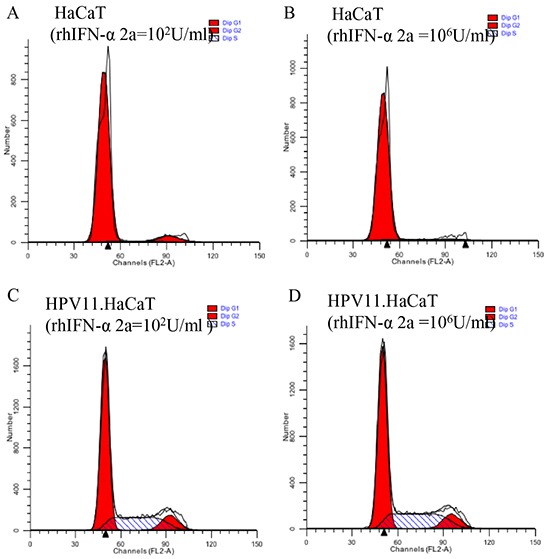
FACS analysis of HaCaT and HPV. HaCaT with rhIFN-α 2a treatment **A&C.** FACS analysis of cell cycles in HaCaT and HPV11.HaCaT cells with 100 U/ml rhIFN-α 2a treatment. **B&D.** FACS analysis of cell cycles in HaCaT and HPV11.HaCaT cells with 10^6^ U/ml rhIFN-α 2a treatment. No significant difference showed in different concentration of rhIFN-α 2a treatment.

**Figure 4:**
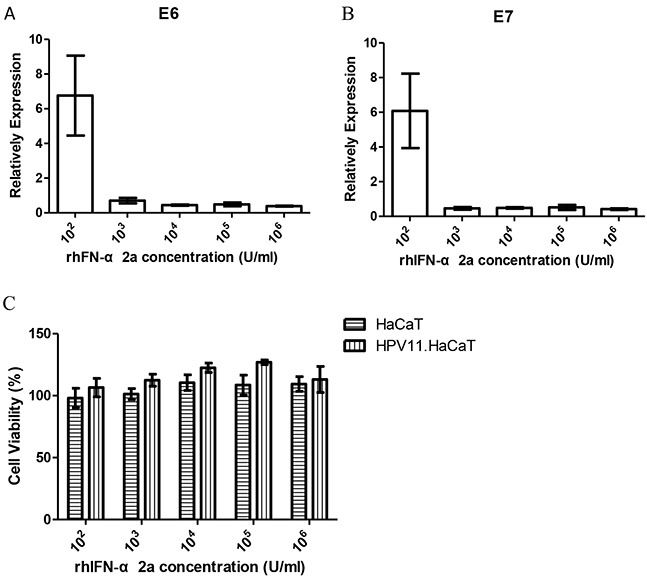
Gene expression difference and cell viability HPV11.HaCaT cells with dose dependent rhIFN-α 2a treatment for 24h **A&B.** After treated for 24h, 1000U/ml or higher concentration rhIFN-α 2a could significantly decrease the expression of HPV11 E6 and E7 mRNA (*P*<0.05). Data are means ± SD from three independent experiments. **C.** Cell viability did not change significantly with the different concentration of rhIFN-α 2a concentration.

RhIFN-α is widely used in treatment of CA in clinical. We used rhIFN-α as the testing agents to assess the practicability of the recombinant cells. FACS analysis for cell cycle showed similar results at the lowest (10^2^U/ml) and the highest (10^6^U/ml) concentration of rhIFN-α 2a treatment. Neither the lowest nor the highest concentration of rhIFN-α 2a showed significant effect on proliferation of both HaCaT and HPV11.HaCaT cells (Figure 3A–3D). The effects of rhIFN-α 2a on expression of HPV11 E6 and E7 mRNA were showed in Figure 4. Low concentration of rhIFN-α 2a (100U/ml) exhibited increased replication of HPV11 E6 and E7 mRNA (*P*<0.05). However, 1000U/ml or higher concentration of rhIFN-α 2a could significantly decrease the expression of HPV11 E6 and E7 mRNA (*P*<0.05). MTT assay showed that rhIFN-α 2a didn't inhibit the proliferation of both HaCaT and HPV11.HaCaT cells (Figure 4C).

### IFN signaling pathway in HPV11.HaCAT cells

**Figure 5:**
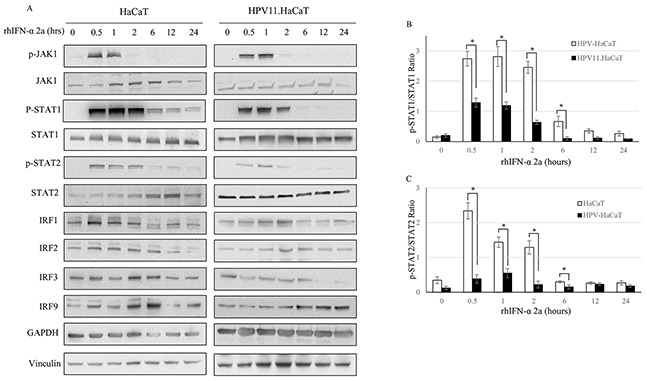
The suppression of JAK-STAT signaling pathway in HPV11.HaCaT Cell line **A.** Western blotting analysis of HaCat and HPV11.HaCaT Cells after rhIFN-α 2a treatment (1000U/ml). **B.** pSTAT1 was significantly decreased in HPV11.HaCaT Cells after IFN-α treatment for 0.5 to 2 hours. **C.** pSTAT2 was significantly decreased in HPV11.HaCaT Cells after rhIFN-α 2a treatment for 0.5 to 2 hours. Data are means ± SD from three independent experiments.

HaCaT cells have the ability to induce immune response. After infected with HPV, host cells were supposed to response to the virus infection. However, in HPV11.HaCaT cells, JAK-STAT pathway might be inactivated. It is hypothesized that the recombinant cells might be in homeostasis with stable virus infection, while the immune response signaling pathway was inactivated (Figure 5). In order to verify the activation of IFN signaling pathway in recombinant cells, we stimulated the cells using an exogenous rhIFN-α. Western Blot test showed that JAK-STAT signaling pathway was activated both in HPV11.HaCaT cells and in HaCaT cells stimulated with 1000U/ml rhIFN-α 2a (Figure 5A). However, p-STAT1 and p-STAT2 expression levels were lower in HPV11.HaCaT cells when compared to in HaCaT cells (Figure 5B&5C).

### The anti-HPV effect EGCG

**Figure 6:**
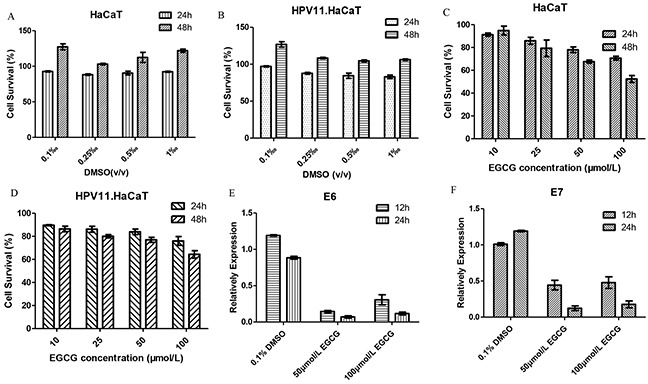
The anti-HPV effect of EGCG in HPV11.HaCaT cells **A&B.** DMSO treatment showed no cytotoxicity for HaCat and HPV11.HaCaT Cells. **C&D.** EGCG treatment affects cell viability for HaCat and HPV11.HaCaT Cells. But cell survival was more than 80% in both cells in 24h. High concentration (100μmol/L) EGCG showed significant inhibition on cells proliferation in 48h as the cell survival decreasing to 60%. **E&F.** After treated for 12 or 24 hours, 50μmol/L and 100μmol/L EGCG could significantly decrease the expression of HPV11 E6 and E7 mRNA (*P*<0.05), while DMSO as solvent didn't show an ability to decrease HPV11 E6 and E7 mRNA expression. Data are means ± SD from three independent experiments.

EGCG is one of the main active component of tea catechins (sinecatechins). EGCG was dissolved in DMSO, and no cytotoxicity of 0.01%-0.1% (v/v) DMSO was detected by MTT assay (Figure 6A&6B). The results of MTT assay showed the effect of EGCG at different concentration groups on proliferation of HPV11.HaCaT cells after treatment for 24h and 48h, respectively. In 24h, at the range of 10 to 100 μmol/L concentration of EGCG, cell survival rates of different dose groups were over the criteria of 80%, while high concentration (100 μmol/L) of EGCG significantly inhibited the proliferation of HPV11.HaCaT cells and HaCaT cells in 48h (Figure 6C&6D). According to the above results, we selected 50 and 100μmol/L concentrations of EGCG for 12 and 24h treatment respectively to conduct HPV11 E6 and E7 mRNA expression tests. The results of real-time PCR showed that EGCG at these two concentration group significant decreased expression of HPV11 E6 and E7 mRNA compared with corresponding DMSO vehicle group (*P*<0.05, Figure 6E&6F). And the inhibiting effect was stronger in 24 hours than 12 hours (*P*<0.05). In 12 hours, 50 μmol/L EGCG group had 15% E6 mRNA expressed, while 100 μmol/L EGCG group was 30% (*P*<0.05). In 24 hours, the expression of HPV11 E6 mRNA had no statistical difference between two EGCG concentration groups (*P*>0.05). Moreover, there was no significant difference of E7 mRNA expression between the two concentration groups at 12 and 24 hours, respectively (*P*>0.05).

## DISCUSSION

HPVs are small DNA virus which is exclusively intraepithelial pathogens and dependent on differentiation status of host cells [4, 5]. Naturally, the viruses first infect the basal layer of epithelium with microtrauma [14]. The viruses and cells replicate simultaneously and the number of copies of the virus genes, mainly early genes, maintains at low levels in basal layer. When basal cells differentiate and migrate to the upper layer, virus genes expression becomes deregulated and the gene copies reach a high level [15]. Unlike other viruses, HPVs do not have blood-borne phase or cause cell death. When infecting cells, HR-HPVs integrate their genes (E5, E6 and E7) into the host cell genome. Different from HR-HPVs, after infecting host cells, LR-HPVs’ genome is an episome in host cells, in a form of plasmid. And that's why we transfected the self-circulated HPV11 genome into HaCaT cells to mimic HPV11 infected epithelium naturally. We recombined the cells and cultured them in medium with low calcium concentration to simulate the infected cells in basal layer. Our results suggest that the recombinant cells containing HPV11 genome proliferated relatively rapidly than HaCaT cells. In fact, unlike HR-HPVs, after infection with LR-HPVs, the lesions are formed with only limited cell proliferation in basal cell layers, because the genomes of LR-HPVs do not accelerate growth of host cells they infect [16].

HPV's early genes involved in replication and pathogenicity of viruses. Our results confirm the expression of several early genes (E1^E4, E5, E6 and E7) of HPV11 in recombinant cells. In our previous study, we cultured the HPV11.HaCaT cells in raft system to mimic the differentiation of epithelium in human. However the raft system was time-consuming with multiple limitations. Recently, we explored to bathe cells in semi suspension medium and culture our recombinant cells in 1.6% methylcellulose. In this semi suspension culture medium, cells tended to be differentiated. We detected the expression of involucrin by immunocytochemistry. Involucrin (IVL) is one of the precursor proteins of keratinocyte cornified envelope that is formed beneath the inner surface of the cell membrane during terminal differentiation [17]. IVL is an important marker gene that serves as a model for understanding the mechanisms that regulate the differentiation process [18]. Our research showed that the expression of HPV11 E6 and E7 mRNA significantly increased in differentiated recombinant cells compared with undifferentiated cells. Also, result of flow cytometry showed the differentiated cells performed stronger proliferation activity. All the above results suggested that HPV11 early genes could normally express in recombinant cells harbouring HPV11genome and the differentiated recombinant cells may imitate the status of virus early genes expression in naturally infected human epithelium.

RhIFN-α and tea catechins are used in treatment of CA. Here, using our recombinant cells we tested anti-HPV11 effects of rhIFN-α 2a and EGCG. The results show rhIFN-α 2a didn't affect proliferation of HPV11.HaCaT cells significantly. But rhIFN-α 2a at ≥1000U/ml of concentration inhibited expression of HPV11 E6 and E7 mRNA significantly. However, we also observed that 100U/ml rhIFN-α 2a exhibited increase of HPV11 E6 and E7 mRNA expression, which suggests that low concentration of rhIFN-α 2a tends to promot replication of HPV11 genes. The chosen concentrations of EGCG did not inhibit proliferation of HPV11.HaCaT cells significantly in 24h, but it decreased expression of HPV 11 E6 and E7 mRNA respectively. In addition, the difference in E6 and E7 mRNA expression in different time may also suggest that HPV 11 gene might activate some bypass feedback to promote replication of the virus gene. The results of rhIFN-α 2a and EGCG indicate that their inhibitory effects on expression of HPV11 E6 and E7 mRNA might be one of the mechanism for anti-HPV11, in addition to immune regulation or antioxidant effect. Meanwhile, these results verified the availability of recombinant cells containing HPV11 genome for testing anti-HPV11 activity.

Whether in forms of episome or integrating in genome of host cells, interaction between HPVs genes and host cells plays an important role in maintaining infection. As an in vitro model, we should take into account if virus genes impact on cellular microenviroment after HaCaT keratinocytes harbouring HPV11 genome. We studied IFN signal pathway in our recombinant cells. It has been known that pathogen recognition receptors (PRRs), such as Toll-like receptors (TLRs), are expressed in normal keratinocyte [19]. Therefore, keratinocytes can sense the pathogens and mediate immune responses after HPVs infection. Activation of TLRs leads to production of type I interferons (IFNs) [20]. The type-I IFNs, including IFN-α and IFN-β, have antiviral, antiproliferative, antiangiogenic, and immunostimulatory properties. They mediate their biological responses through the STAT family of transcription factors [21, 22]. When infected with HPVs, the HPV oncogenes interact with components of IFN signaling pathways and inhibit the IFN-α-mediated signaling. For example, HPV16 E7 interfered with intermediate IFN-mediated signals by physically associating with IRF-1, inhibiting IRF-1-mediated activation of the IFN- promoter for recruitment of histone deacetylase to the promoter, thereby preventing transcription [12]. The E6 protein of HPV16 also targeted the interferon pathway. E6 prevented ligands binding to the the IFN receptor and inhibited phosphorylation of STAT1, and STAT2, impairing JAK-STAT activation and therefore specifically inhibiting IFN-α-mediated signaling [11].

Considering recombinant cells being in a state of homeostasis after culture and propagation, we speculated the JAK/STAT signaling pathway in HPV11.HaCaT cells might be suppressed. Thus, we used rhIFN-α 2a as an exogenous stimulator to test the integrity of IFN signaling pathway in our recombinant cell. The results showed that IFN signaling pathway in recombinant cells was activated after stimulation with rhIFN-α 2a, but the levels of p-STAT1 and p-STAT2 proteins expression significantly decreased compared with the HaCaT cells during the first 2h of stimulation, and the difference between the two cells was not obvious when extending stimulation until 24h. Take the results of RT-PCR of into consideration, HPV11 E6 and E7 mRNA expression being significant decrease with rhIFN-α 2a treating recombinant cells for 24h, it suggests that when stimulated by IFN-α, the recombinant cells present normal type-I IFN signal cascade as the normal HaCaT cells, and the activation of IFN-α signal pathway relates to levels of HPV11 E6 and E7 expression.

According to above results, we further confirmed the integrity of HPV11 early genes expression and growth characteristic of recombinant cells as an in vitro model. Using rhIFN-α 2a and EGCG as testing agents, we also verified its availability as a tool for testing anti-HPV11 activities. The study data of IFN JAK/STAT pathway imply the impact on cellular microenviroment with inducting HPV11 genome.

However, the current study has several limitations. In this study, we tested rhIFN-α 2a and EGCG as well as the IFN pathway in undifferentiated recombinant cells. In clinic, CA patients’ lesion are highly differentiated and copies of HPV genes are at high level, which has difference from our cells. However, what is challenging in CA treatment is the recurrence after lesion destruction, which is mainly caused by the remaining virus in basal layer. Therefore, our recombinant cell model still has its clinical significance. Moreover, we are working on a differentiated state of HPV11.HaCaT to mimic all the state of pathogenic epithelium and explore interaction between HPV11 genome and host cells using our recombinant cells.

In conclusion, we verified the integrity, functionality and practicability of the recombinant cells which were supposed to be used as anti-HPV screening model. Comparing to HaCaT cell line, alternation of characteristics in HPV11.HaCaT cells, including cell growth and cell cycle as well as JAK/STAT signaling pathway, have been fully addressed. Moreover, our anti-HPV11 screening model demonstrated that EGCG inhibit HPV11 oncogenes expression significantly.

## MATERIALS AND METHODS

### Transfection of HPV11 genome to HaCaT cells

The HaCaT cells were cultured as previously described [8]. The pBR322.HPV11 plasmid [12,294 base pairs (bp)] from *Escherichia coli* (ATCC No. 45151, ATCC, USA) was extracted and purified, following which the plasmid was digested with BamHI enzyme (Promega, USA) to release the linear full-length HPV-11 genome. The linear genome was then self-circulated with T4 DNA ligase (Invitrogen, USA). After the above steps, the circularized HPV 11 DNA and pTK-neo DNA (Novagen, USA) were transfected into HaCaT cells. After selection with G418 (Sigma, USA), the remaining cell colonies were pooled as a cell population, which was named HPV11.HaCaT [8].

### Cell growth curve

The HaCaT and HPV11.HaCaT cells were cultured as described previously [8]. The cells were collected, resuspended with new fresh medium and subsequently counted. After that both HaCaT and HPV11.HaCaT cells were inoculated into 21 culture bottles, where every bottle contained 5×10^4^ cells. 3 bottles of each cells were counted every 24 hours for 7 days. Growth curves were plotted to visualize the cell counts changes with the extension of culture time.

### Immunofluorescence

HPV11.HaCaT cells were cultured overnight on glass slides, which were in 3 cm petri dishes. The cultures were rinsed three times with PBS and fixed in 4% paraformaldehyde solution. 1ml 30% triton-X-100 was added in 299ml TBS to compound scrubbing solution. Subsequently, they were washed and then blocked by goat serum for 1h at room temperature. Then incubated overnight at 4°C in anti-HPV11 E7 antibody (1:250 dilution in blocking buffer; Abcam, USA) or anti-involucrin antibody (1:200 dilution in blocking buffer; Sigma-Aldrich, USA), washed three times for 5 min each time, followed by incubating in goat anti-mouse IgG-conjugated with Alex Fluor 488 (1:400 dilution in PBS; Beyotime, China) for 1 h at 37°C in dark. DAPI solution (3 μg/mL in PBS; Beyotime, China) was used for nuclear staining. Samples was observed under a laser scanning confocal microscope (Olympus, Japan). In the fluorescent images, cytoplasm displayed as green fluorescence and the nucleus displayed as blue.

### Differentiation of HPV11.HaCaT in semisolid media

The HPV11.HaCaT cells were suspended in 1.6% methylcellulose to induce differentiation. The methylcellulose solution was prepared by adding half of the final volume of DMEM to autoclaved dry methylcellulose (Sigma, USA) and heating the mixture in a 60°C water bath for 20 min. The remaining DMEM was added, and the mixture was stirred at 4°C overnight until clear. After harvested with trypsin digestion, HPV11.HaCaT cells were resuspended in 1 ml of the methylcellulose, and added dropwise to a 6 cm petri dish containing 15 ml of 1.6% methylcellulose. Cells were stirred with a pipette and incubated at 37°C in a humidified 5% CO_2_ incubator for 24 hours. Cells in methylcellulose were harvested before reaching 80% confluence. Samples were subsequently subjected to fluorescence-activated cell sorting (FACS) and extract total RNA for real-time PCR.

### FACS analysis

HaCaT and HPV11.HaCaT cells were digested with trypsin without EDTA. Wash with PBS, and then fix cells with 70% ice cold ethonalto. The samples, stored at −20°C, were tested by fluorescene-activated cell sorting (FACS).

### MTT assay

RhIFN-α 2a (Peprotech, USA) was dissolved in DMEM. Five groups were designed and the concentration ranged from 10^2^ to 10^6^ U/ml. EGCG (Sigma, USA) was dissolved in dimethylsulfoxide (DMSO; Sigma, USA) at 100 mM and stored at −20°C before use. Before experiments, diluted the EGCG storage solution with DMEM and got four different concentration groups as 10, 25, 50, 100μmol/L. The final concentration of DMSO in culture medium was 0.01-0.1% (v/v). In all experiments control cultures were made up of medium, DMSO or DMEM. The effect of rhIFN-α and EGCG on cell proliferation was examined by MTT assay. HaCaT and HPV11.HaCaT cells were seeded in 96-well plates and cultured overnight. Then rhIFN-α 2a, EGCG and DMSO (vehicle control) were added at the above concentrations. Every treatment was repeated for three times. After 24 and 48 hours, 20 μL of 5 mg/mL MTT solution (Sigma, USA) was added to each well and incubated for 4 hours at 37°C. The MTT formazan crystal was then dissolved in 150μL DMSO, and the absorbance was measured by a microplate reader (Thermo, USA) at a wavelength of 550 nm.

### Nested PCR and real-time PCR

HaCaT and HPV11.HaCaT cells were seeded in six-well plates (1×10^6^ cells each well) and grown to about 80% confluence before harvest. Total cellular RNA was extracted using TRIZOL reagent (Invitrogen, USA), and cDNA was synthesized using the Reverse Transcription System (Promega, USA). The products were amplified by nested primer sets and two 30-cycle PCR rounds with the outer primer and inner primer, respectively, to detect the HPV-11 E1^E4 spliced transcript. The primers are described in Supplementary Table S1. Ten microliters of cDNA were used for the first 30-cycle reaction, and 2 μL of the first PCR product was used for a second 30-cycle round of PCR. The temperature file is as follows: first, 2 min at 94°C, then 30 cycles of 30 s at 94°C, 30 s at 60°C, and 55 s at 72°C, with a final extension of 10 min at 72°C. Normal HaCaT cells without HPV- 11 DNA were used as a blank control.

According to the result of MTT assay, 50 and 100μmol/L EGCG and all the 6 concentrations of IFN were chosen to treat the HPV11.HaCaT cells. After treated for 6, 12 and 24h, total RNA was isolated and cDNA was synthesized as mentioned above. The primers for real-time PCR are described in Table 1. 10 μL of 2 × SYBR Green PCR Master Mix (ABI, USA), 1.5μL of cDNA, 0.8 μL of primers (F/R) and 6.9μL of nuclease free water were contained in a final reaction volume of 20μl. The temperature file was as follows: 50°C for 2 min, 95°C for 2 min followed by 40 cycles of 95°C for 15 s, 58°C for 15s, 72°C for 1min, and the melting curve was analyzed at the end to ensure the product specificity. Each sample was performed in triplicate. The data were analyzed with the comparative CT (ΔΔCT) method and the amount of target genes (2^ΔΔCT^) was obtained by normalizing to an endogenous reference (β-actin) and compared to the respective control group. Relative levels of mRNA were determined by RT-PCR using ABI Prism 7300 real time PCR system (Applied Biosystems, USA).

**Table I:** Oligonucleotide primers used in PCR

Gene Target	Primer	Sequence (5′-3′)
E1^E4	First PCR forward	5′-TAC AAG ACC TTT TGC TGG GCA CA-3′
(nested PCR)	First PCR reverse	5′-AAA GGC AGG AAA ATA GCA CAC AC-3′
	Second PCR forward	5′-ATA TTG TGT GTC CCA TCT GCG -3′
	Second PCR reverse	5′-CAG CAA TTT GTA CAG GCA CTA C-3′
β-actin	First PCR forward	5′-GAT GAC CCA GAT CAT GTT TG-3′
(nested PCR)	First PCR reverse	5′-GGA GCA ATG ATC TTG ATC TTC -3′
	Second PCR forward	5′-AAC ACC CCA GCC ATG TAC GTT G-3′
	Second PCR reverse	5′-ACT CCA TGC CCA GGA AGG AAG G-3′
E5	forward	5′-AGT GCC TGT ACA AAT TGC TGC-3′
	reverse	5′-AGG CAG GAA AAT AGC ACA CA-3′
E6	forward	5′-TAC CTG TGT CAC AAG CCG TT-3′
	reverse	5′-CAG CAG TGT AAG CAA CGA CC-3′
E7	forward	5′-GTG GAC AAA CAA GAC GCA CA-3′
	reverse	5′-CTGT GCA CTC CAC AAC CAG T-3′
β-actin	forward	5′-AGC GAG CAT CCC CCA AAG TT-3′
	reverse	5′-GGG CAC GAA GGC TCA TCA TT-3′

### Western blot

HaCaT and HPV11.HaCaT cells were treated with 1000U/ml rhIFN-α 2a for 0.5, 1, 2, 6, 12 and 24 hours respectively. Then cells were washed with PBS and lysed in a buffer containing: 50 mM Tris-Cl (pH 7.4), 1 mM EDTA, 150 mM NaCl, 0.1% SDS, 1% Triton X-100, 1% sodium deoxycholate, 1% NP-40, and 1 mM PMSF. Protein concentration was determined with BCA protein assay kit. Then equal amount of protein was subjected to electrophoresis in a 12% polyacrylamide-SDS gel and then transferred onto a NC membrane using wet transfer system (Bio-Rad, USA). The primary antibodies were rabbit polyclonal GAPDH (1:300; Goodhere, China), p-Stat1 (1:600; CST, USA), Stat1 (1:600; CST, USA), p-Stat2 (1:600; CST, USA), Stat2 (1:600; CST, USA), p-JAK1 (1:600; CST, USA), JAK1 (1:600; CST, USA), IRF1 (1:200; Santa Cruz, USA), IRF2 (1:200; Santa Cruz, USA), IRF3 (1:500; CST, USA) and IRF9 (1:500; Abcam, USA). SuperSignal West Pico Chemiluminiscence Substrate (Pierce Biotechnology, USA) was used for detection antibodies and membranes were scanned for the quantification using Bio-Rad Quantity One software. All target signals were normalized with the GAPDH in the same sample.
